# Hemato-Oncology Care During and After the COVID-19 Pandemic: Changes in Treatment Pathways, Patient Flow, and Durable Organizational Adaptations

**DOI:** 10.7759/cureus.111331

**Published:** 2026-06-22

**Authors:** Paul C Toboltoc, Ioana Gagiu, Alex L Lukusa, Adela Vekony

**Affiliations:** 1 Pathology, County Clinical Emergency Hospital of Sibiu, Sibiu, ROU

**Keywords:** cancer care delivery, covid-19, hematologic malignancies, hemato-oncology, organizational adaptation, patient flow, telemedicine, treatment pathways

## Abstract

The COVID-19 pandemic disrupted cancer care at every level of the clinical pathway, but its effect on hemato-oncology was distinctive because patients with hematologic malignancies often require urgent diagnosis, frequent hospital visits, transfusion support, intensive therapy, transplantation, cellular therapy, and infection-sensitive follow-up. This narrative review examines the pandemic as a disruption of hemato-oncology care delivery rather than only as a source of excess infection-related morbidity. It synthesizes evidence on diagnostic access, patient flow, treatment prioritization, infection-control circuits, day-hospital organization, inpatient access, telemedicine and hybrid follow-up, supportive care, vaccination, antiviral pathways, and high-complexity care delivery. The review distinguishes between temporary crisis restrictions, harmful diagnostic and therapeutic delays, and durable organizational adaptations that may remain relevant after the acute pandemic period. Evidence from cancer-service disruption studies, hemato-oncology outcome registries, telemedicine cohorts, and European hematopoietic cell transplantation activity surveys indicates that hemato-oncology care pathways were reorganized and selectively redirected during the pandemic, while selected components recovered through structured triage, protected circuits, remote review of stable patients, and individualized timing of transplantation and cellular therapy. The post-pandemic priority is not to preserve restrictive crisis practice, but to retain the organizational discipline generated by the crisis: protected diagnostic capacity, risk-stratified access, selective hybrid care, rapid infectious-risk assessment pathways, disciplined day-hospital scheduling, and reserved in-person capacity for unstable, procedure-dependent, or curative-intent patients. These adaptations should be maintained only insofar as they improve access, safety, and continuity of care without normalizing delayed diagnosis or undertreatment of aggressive disease.

## Introduction and background

The COVID-19 pandemic rapidly disrupted oncology systems, affecting diagnostic pathways, outpatient activity, elective admissions, treatment delivery, supportive care, and hospital resources [1,2].

For hemato-oncology, the disruption was particularly complex because patients with hematologic malignancies (HMs) often combine disease-related immune dysfunction, treatment-induced cytopenias, transfusion or venous-access needs, and repeated contact with outpatient, day-hospital, inpatient, and procedural services. In selected aggressive entities, these vulnerabilities coexist with a narrow therapeutic window in which diagnostic or therapeutic delay can rapidly worsen clinical outcome [3-6].

Before the pandemic, routine hemato-oncology care was largely hospital-centered, relying on in-person evaluation, same-site laboratory review, imaging or bone marrow procedures, day-hospital chemotherapy or immunotherapy, and inpatient admission for induction treatment, febrile neutropenia, transfusion support, transplantation, or severe toxicity [5,6]. This model supported close supervision, rapid escalation, and procedural coordination within specialist centers, but it also generated frequent hospital exposure for a treatment-dependent and immunologically fragile population, a weakness that became evident once SARS-CoV-2 transmission affected clinical environments [3,4,6].

Because the pandemic did not eliminate the need for hemato-oncology care, clinicians had to make difficult decisions, often with limited resources. They had to identify which patients required immediate assessment or treatment, which cases could safely be postponed, which interventions had curative or high clinical value, and which hospital visits were truly necessary rather than part of routine follow-up [5,6].

The operational response, although broad and uneven across institutions, included pre-visit screening, SARS-CoV-2 testing before selected admissions or intensive treatments, separated infectious and non-infectious circuits, reduced waiting-room density, disciplined day-hospital scheduling, modified thresholds for urgent review, and local laboratory testing where feasible [5,7]. In parallel, telephone and video consultations were expanded for selected encounters that did not require physical examination, procedures, treatment administration, or urgent clinical assessment [8].

Several of these changes were temporary and restrictive, and some became harmful when fear of infection, reduced diagnostic capacity, movement restrictions, or overloaded hospitals delayed first presentation or definitive diagnosis [2,9,10].

These changes also exposed pre-existing organizational vulnerabilities, including routine hospital visits for stable chronic hematologic disease, fragmented day-hospital scheduling, avoidable travel for prescription renewal or laboratory review, limited use of structured pre-visit triage, and insufficient separation between immunocompromised patients and patients with respiratory symptoms [7,8,11].

The central question after the pandemic is therefore not whether pandemic restrictions should continue, but which crisis-driven adaptations proved durable enough to become organizational tools and which should be deliberately abandoned. This narrative review summarizes changes in treatment delivery, patient flow, and care organization for adult patients with HMs during and after the COVID-19 pandemic, with particular emphasis on differences between pre-pandemic, pandemic, and post-pandemic care, published data that illustrate disruption, risk, and recovery, and practical adaptations that may remain useful for hematology units, oncology day hospitals, transplant programs, and outpatient follow-up pathways. Accordingly, this article should be read as a narrative service-delivery synthesis rather than as a disease-specific therapeutic review or a systematic evidence map.

Previous reviews have often addressed SARS-CoV-2 outcomes, vaccine response, infection management, or disease-specific treatment modifications in patients with HMs [4-6,12,13]. By contrast, this review approaches the pandemic as a disruption of hemato-oncology care delivery itself. Its main contribution is to integrate diagnostic access, patient flow, treatment prioritization, day-hospital organization, inpatient access, transplantation and cellular therapy logistics, hybrid follow-up, and post-pandemic infection-management pathways into a single service-delivery framework, distinguishing temporary crisis restrictions from adaptations that may reasonably persist after the acute pandemic period.

Methodology

This paper was designed as a narrative review, not as a systematic review or meta-analysis, because its purpose was to synthesize several overlapping clinical and organizational domains rather than to estimate a single pooled effect. The methodological structure was therefore centered on transparent source selection, thematic grouping, and critical narrative integration, in keeping with the quality principles described by the Scale for the Assessment of Narrative Review Articles (SANRA) [14]. A focused literature search was performed in PubMed/MEDLINE and Google Scholar, supplemented by targeted consultation of official guidance, registry reports, and institutional publications relevant to hemato-oncology care, cancer-care disruption, telemedicine, transplant activity, supportive care, vaccination, antiviral treatment, and post-pandemic service recovery. The final search update was performed in May 2026.

To make the narrative process more transparent and traceable, the search strategy was organized around prespecified thematic axes: cancer-care disruption and diagnostic delay, COVID-19 outcomes in HMs, treatment prioritization, telemedicine and hybrid follow-up, day-hospital and inpatient patient flow, hematopoietic cell transplantation (HCT) and cellular therapy, supportive care, vaccination, antiviral pathways, and post-pandemic service recovery. The search was iterative, but source retention required direct relevance to at least one of these axes rather than general relevance to COVID-19 alone.

Search terms were combined using disease-, care-delivery-, and pandemic-related concepts, including “COVID-19”, “SARS-CoV-2”, “hematologic malignancies”, “haematological malignancies”, “hemato-oncology”, “leukemia”, “lymphoma”, “multiple myeloma”, “patient flow”, “cancer care disruption”, “telemedicine”, “virtual care”, “hematopoietic cell transplantation”, “cellular therapy”, “day hospital”, “triage”, “supportive care”, “vaccination”, and “antiviral treatment”. Publications from January 2020 to May 2026 were prioritized, while selected pre-pandemic or non-COVID sources were retained when they clarified baseline care models, narrative review methodology, or the interpretation of treatment delay. Sources considered relevant included multicenter registry studies, systematic reviews, meta-analyses, guideline or consensus documents, institutional cohort studies, health-system reports, and single-center experiences when they provided numerical data or clinically relevant descriptions of pathway redesign. Sources were not retained when they focused exclusively on virology without implications for care organization, addressed pediatric hemato-oncology without transferable adult pathway information, consisted of opinion pieces without identifiable clinical or organizational evidence, or duplicated data already captured in larger registries or consensus statements.

Example search combinations included (“COVID-19” OR “SARS-CoV-2”) AND (“hematologic malignancies” OR “haematological malignancies” OR leukemia OR lymphoma OR myeloma) AND (“patient flow” OR triage OR telemedicine OR transplantation OR “cellular therapy” OR “cancer care disruption”). A second group of searches combined (“COVID-19” OR “SARS-CoV-2”) AND (“hematopoietic cell transplantation” OR “stem cell transplantation” OR “CAR T-cell”) AND (activity OR delay OR prioritization OR guidance).

During source selection, priority was given to publications that provided quantitative disruption signals, clinically actionable recommendations, registry-level outcome data, real-world service-flow descriptions, or clear post-pandemic relevance for care delivery. General oncology or health-system studies were retained only when hemato-oncology-specific evidence was unavailable or when they helped interpret broader service-level changes relevant to hematology practice.

Rather than being extracted for statistical pooling, information from each source was grouped according to population, country or setting, care domain, quantitative disruption signal, clinical adaptation, and post-pandemic relevance. Particular attention was given to pre-pandemic, pandemic, and post-pandemic contrasts, including diagnostic or treatment disruption, telemedicine uptake, hospitalization patterns, transplant activity, vaccination-era changes in risk, and the transition from emergency restriction to selective post-pandemic retention. The synthesis used narrative evidentiary weighting, in which direct hemato-oncology evidence was prioritized whenever available, while broader cancer-care or health-system evidence was used only when it clarified service disruption, telemedicine uptake, transplant activity, or continuity of care. Direct evidence from patients with HMs and transplant registries was interpreted separately from general oncology or public-health data.

Source weight was interpreted narratively according to study design, population, setting, and proximity to the review question. Numerical data were transcribed only from published articles, registry reports, health-system reports, or consensus and guideline documents, and were used descriptively for pathway-level interpretation.

The review focuses on adult patients with HMs, including acute leukemias, aggressive and indolent lymphomas, chronic lymphocytic leukemia (CLL), multiple myeloma and related plasma cell disorders, myeloid neoplasms, patients undergoing HCT, and patients receiving cellular or immune-based therapies, including chimeric antigen receptor T-cell (CAR T-cell) therapy. Pediatric hemato-oncology, purely virological aspects of SARS-CoV-2 infection, and vaccine immunogenicity were considered only when they affected patient flow, treatment delivery, or post-pandemic care organization.

## Review

Hemato-oncology care before the pandemic

Before the pandemic, routine hemato-oncology care was organized primarily around clinical proximity, repeated assessment, rapid access to laboratory data, procedural dependence, and hospital-based coordination rather than exposure minimization [5,6]. Patients with aggressive lymphoma, acute leukemia, or newly symptomatic plasma cell neoplasms commonly entered care through emergency departments (EDs), inpatient hematology wards, or urgent outpatient routes [5,6]. In acute leukemia in particular, diagnostic and treatment pathways remained highly time-sensitive, while the broader diagnosis of HMs often required morphology, flow cytometry, immunohistochemistry, cytogenetics, molecular testing, imaging, and clinical staging, which explains why disruptions in diagnostic logistics could affect treatment timing [5,15].

Patients with indolent lymphoma, CLL, chronic myeloid leukemia (CML), low-risk myelodysplastic syndromes (MDS), or stable myeloproliferative neoplasms (MPNs) could often be monitored without immediate therapy [5]. Yet even in these groups, follow-up frequently remained anchored in periodic hospital attendance before the pandemic, although some encounters consisted mainly of laboratory review, prescription continuity, symptom screening, or reassurance visits that could later be redirected into selective hybrid care models [8,11,16]. This in-person model had clear advantages before COVID-19: complex decisions were concentrated in specialist centers, complications could be detected through direct examination, transfusions and infusions were available on site, and patients with unstable disease could move rapidly from outpatient assessment to admission [5-7]. Its weakness became evident when repeated hospital contact itself became hazardous for a population in which early COVID-19 studies reported substantial mortality. Passamonti et al. reported 37% mortality in the Italian Hematology Alliance cohort, while, in a systematic review and meta-analysis of 3,377 patients with HMs and COVID-19, Vijenthira et al. estimated 34% mortality among adults with HMs [3,4].

Telemedicine and remote monitoring existed before the pandemic, but they were not yet structural components of routine hemato-oncology care [8,11,16]. Lloyd et al. emphasized that telemedicine in HMs was still developing and that hematology practices had been slower to adopt remote care because traditional models relied heavily on interdisciplinary, regionally organized, and procedure-dependent care [16]. Suleman et al., in a scoping review of virtual care in HMs, found that published studies covered lymphoma, CML, transplantation, MDS, MPNs, and AML, and concluded that virtual care appeared feasible and was generally associated with high patient satisfaction, although provider concerns and technical barriers remained relevant [11]. The pandemic therefore moved telemedicine from a peripheral adjunct toward a selective operational tool, particularly for encounters based on laboratory review, symptom screening, treatment discussion, or follow-up rather than physical examination or procedures [8,11,16].

Early pandemic disruption

The earliest pandemic waves disrupted cancer care through direct and indirect mechanisms: hospitals redirected resources toward COVID-19, screening and routine diagnostic procedures were suspended or reduced, patients avoided healthcare settings because of infection fear, movement restrictions limited access, and diagnostic capacity was unevenly restored across institutions [1,2,17]. Richards et al. described this disruption across the cancer-care spectrum, while later global evidence confirmed that the first pandemic year was associated with substantial reductions in screening, diagnosis, diagnostic procedures, and treatment activity [1,2].

The magnitude of this disruption was measurable. In a global systematic review and meta-analysis of 245 studies from 46 countries, Shah et al. estimated reductions during the first pandemic year in cancer screening participation, cancer diagnoses, diagnostic procedures, and treatment activity of 39.0%, 23.0%, 24.0%, and 28.0%, respectively [2]. Although Shah et al.’s estimates were not restricted to HMs, they describe the system-level pressure under which hemato-oncology units also operated [2]. This interpretation is supported by hemato-oncology-specific service data from Yerdenova et al., who retrospectively analyzed 6,763 hemato-oncology hospitalizations across two tertiary centers in Almaty between March 1, 2019, and February 28, 2022 [18]. Compared with 2019-2020 and 2021-2022, the pandemic year 2020-2021 was associated with fewer discharges, 2,047 versus 2,387 and 2,081, a higher death proportion, 3.5% versus 3.2% and 2.6%, and more frequent ambulance admissions than in 2019-2020, 19.0% versus 14.8% [18]. The same study also reported a 12.4% increase in transfers from other hospitals to the two hemato-oncology centers during 2020-2021, supporting the interpretation that ordinary referral routes were disrupted and that patients more often reached specialist care through urgent or indirect pathways [18].

For hemato-oncology, diagnostic delay had a different clinical meaning depending on disease tempo, because suspected acute leukemia, aggressive lymphoma, and indolent lymphoproliferative disease do not tolerate postponement in the same way [5,15]. Broader oncology studies support the general principle that delay can worsen outcomes, particularly when the delayed step is diagnostic confirmation or first curative-intent treatment [9,10,19]. In a systematic review and meta-analysis of cancer treatment delay, Hanna et al. found that even a four-week delay in treatment was associated with increased mortality across surgical, systemic therapy, and radiotherapy indications for seven cancers [19]. Maringe et al. and Sud et al. further modeled the survival impact of pandemic-related diagnostic or referral delays in England, supporting the need to protect time-sensitive cancer pathways during system-wide disruptions [9,10]. In hemato-oncology, this principle is especially relevant when the delayed event is a lymph node biopsy in rapidly enlarging disease, a marrow examination in suspected acute leukemia, or a staging assessment before treatment of aggressive lymphoma [5,15].

These disruption signals support the interpretation that delayed access could compress subsequent care entry. In hemato-oncology, this may mean that patients reach specialist services with less time available for orderly staging, pathology review, molecular testing, comorbidity assessment, and treatment planning, particularly when ordinary referral routes are disrupted or urgent pathways become more prominent [18,20]. Yerdenova et al. provide hemato-oncology-specific support for this interpretation by showing altered access routes in Almaty, including more frequent ambulance admissions and increased transfers from other hospitals during 2020-2021 [18]. Hemato-oncology requires protected diagnostic capacity for complete blood counts (CBCs) with urgent smear review, bone marrow biopsy and aspirate, flow cytometry, lymph node biopsy, imaging, transfusion assessment, and molecular or cytogenetic testing, because delayed diagnosis may not simply postpone treatment, but may alter the clinical baseline from which treatment begins [5,6,15].

**Figure 1:**
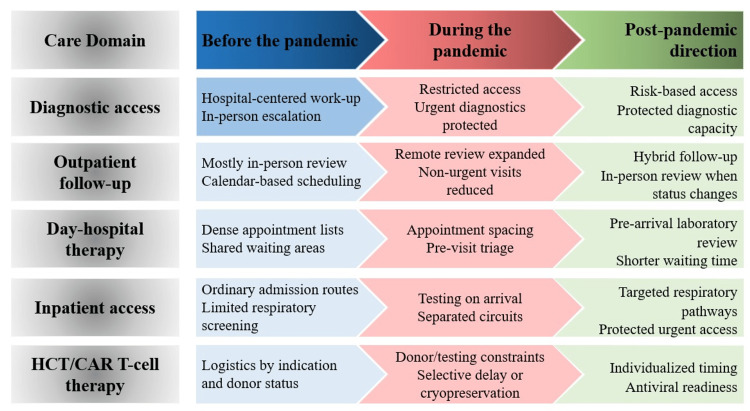
Evolution of hemato-oncology care pathways before, during, and after the COVID-19 pandemic. The figure represents an original conceptual narrative synthesis created by the authors for this review using Microsoft PowerPoint 2016 for Windows (Microsoft Corp., Redmond, WA) and is not intended to serve as a validated care algorithm. HCT, hematopoietic cell transplantation; CAR T-cell therapy, chimeric antigen receptor T-cell therapy

Figure 1 summarizes the proposed before-during-after framework derived from the narrative synthesis, showing that the key structural change was not the disappearance of hospital-based hemato-oncology care, but its redistribution toward protected diagnostic access, controlled day-hospital scheduling, selective remote monitoring, and urgent in-person routes for unstable or procedure-dependent patients.

Clinical vulnerability and changing COVID-19 outcomes in patients with HMs

Patient-flow reorganization was justified by a clinical risk profile that became apparent early in the pandemic. Many patients with HMs had disease-related immune dysfunction before treatment, and many received therapies that deepened immune deficiency, including anti-CD20 antibodies, corticosteroids, purine analogues, proteasome inhibitors, Bruton tyrosine kinase inhibitors (BTK inhibitors), intensive cytotoxic chemotherapy, transplantation, or cellular therapy [5]. Early outcome data confirmed the clinical relevance of this vulnerability: Passamonti et al., through the Italian Hematology Alliance on COVID-19, reported 37% overall mortality among 536 adult patients with HMs and COVID-19, while, in a systematic review and meta-analysis of 3,377 patients, Vijenthira et al. estimated a 34% risk of death among adults with HMs and COVID-19 [3,4].

These early outcome data explains why hemato-oncology units adopted conservative infection-control measures and exposure-reduction strategies during the first pandemic waves [3,4]. Subsequent data, however, showed that risk was dynamic rather than fixed. In the EPICOVIDEHA registry, Salmanton-García et al. analyzed 8,767 COVID-19 cases in patients with HMs from 152 centers in 41 countries and reported a reduction in overall mortality from 29% to 4% between 2020 and 2022, although hospitalization and ICU admission remained associated with worse outcomes [21]. Similarly, in the HEMATO-MADRID COVID-19 study, Martínez-López et al. evaluated 1,818 adult patients with HMs across pandemic phases and found reduced severe illness during the Omicron period, particularly among vaccinated patients, while older age, comorbidities, CLL, hospitalization, and ICU admission remained markers of poor outcome [22].

Registry data from the ASH Research Collaborative Data Hub add another quantitative layer to the early risk signal. Wood et al. reported 250 patients with hematologic malignancies and COVID-19 from 74 international sites and observed 28% overall mortality, with worse outcomes among older patients, those with advanced disease or limited prognosis, and those for whom ICU-level support was not pursued [23]. Read together with the systematic review and meta-analysis by Vijenthira et al., which estimated 34% mortality among adults and 39% mortality among hospitalized adults with HMs and COVID-19, these registry findings support the early use of shielding, screening, route separation, and visit-reduction strategies as responses to a demonstrable risk rather than as nonspecific institutional caution [4,23]. The same level of universal restriction is no longer supported in the same way after changes in population immunity, vaccination, antiviral availability, variant virulence, and clinical experience [21,24]. Targeted protection, however, remains necessary because patients with HMs, especially those with B-cell malignancies, recent B-cell-depleting therapy, cellular therapy, transplantation, uncontrolled disease, or profound immunosuppression, continue to represent a clinically fragile population [12,13,24,25].

Prolonged viral replication was another distinctive feature of this population and had direct implications for isolation, treatment timing, and post-pandemic infectious-risk pathways. Garcia-Vidal et al. studied hospitalized patients with HMs and COVID-19 in Barcelona and documented prolonged SARS-CoV-2 replication in 17 of 67 evaluable patients, corresponding to 25.4% of those assessed after three weeks and 17.3% of hematologic patients alive beyond three weeks [25]. In multivariate analysis, lymphoma, hypogammaglobulinemia, and prior chemotherapy were independently associated with prolonged viral replication, while rituximab exposure was discussed as biologically relevant in the context of impaired B-cell-mediated antiviral response [25].

Treatment prioritization

The central therapeutic question during the pandemic was whether reducing exposure to SARS-CoV-2 justified altering cancer treatment. In hemato-oncology, the answer was disease-specific and intent-specific: when clinically indicated, curative or life-saving therapy for acute leukemia, aggressive lymphoma, advanced symptomatic Hodgkin lymphoma, high-risk myeloma presentations, severe marrow failure, or rapidly progressive disease generally had to proceed, whereas observation, delayed initiation, or less frequent monitoring could be appropriate for stable indolent disease, low tumor burden, or follow-up situations in which immediate intervention was unlikely [5,6,15]. The European Society for Medical Oncology-European Hematology Association (ESMO-EHA) interdisciplinary consensus led by Buske et al. guided clinical decision-making by addressing COVID-19 diagnosis, treatment, mitigation strategies, and the management of specific HMs. These recommendations emphasized that treatment should not be cancelled indiscriminately, but should be tailored to the individual patient according to disease aggressiveness, expected treatment benefit, infection risk, and patient-specific vulnerability [5]. This approach was consistent with broader hematology guidance and disease-specific recommendations [6,26,27]. Isidori et al. emphasized the need to continue effective and potentially curative therapy while reducing exposure, while EBMT acute leukemia guidance from Brissot et al. aimed to minimize ICU need without compromising the patient’s chance of receiving adequate treatment [6,26,27].

In acute leukemia, the potential consequences of delay were particularly serious, because diagnostic and therapeutic postponement could increase the risk of uncontrolled marrow failure, infection, bleeding, leukostasis, transfusion dependence, or early mortality [15,27]. Practical recommendations during COVID-19 therefore emphasized continuing urgent treatment, selecting lower-intensity or outpatient-compatible regimens when clinically reasonable, screening before admission, and reducing exposure during aplasia without allowing infection-control concerns to erase the curative objective in fit patients [15,27]. In aggressive lymphoma, similar logic applied: diagnostic biopsy, staging, and first-line therapy remained time-sensitive, while supportive care, same-day coordination of investigations, telephone toxicity checks, and minimized non-essential visits became tools for reducing exposure without compromising the therapeutic backbone [5,6]. By contrast, in CLL, indolent lymphoma, smoldering plasma cell disorders, stable CML, or selected lower-risk myeloid disorders, the pandemic reinforced principles that already existed before COVID-19 but were not always applied with equal discipline: asymptomatic patients without established treatment indications should be observed rather than treated, laboratory monitoring can often be decentralized, and hospital review should be scheduled according to clinical need rather than habit [5,6]. This was not a reduction in care, but a clearer separation between medical surveillance and hospital attendance [8,11,16].

Disease-specific care implications

The practical value of the pandemic experience becomes clearer when care routes are interpreted by disease tempo rather than by the generic label of hemato-oncology. Acute leukemia requires protected diagnostic and therapeutic channels because marrow failure, leukostasis, bleeding risk, infection, transfusion dependence, or early mortality make delay clinically consequential [15,27]. Aggressive lymphoma and other rapidly progressive HMs similarly require timely biopsy, staging, and curative-intent treatment when clinically indicated [5]. By contrast, indolent lymphoma, CLL, CML, and selected chronic myeloid neoplasms or plasma cell disorders may tolerate hybrid monitoring when the patient is stable, laboratory trends are interpretable, and thresholds for in-person escalation are explicit [5,11]. Plasma cell disorders occupy an intermediate position, because symptomatic myeloma, renal impairment, hypercalcemia, spinal cord compression, infection, or severe cytopenias require rapid in-person assessment, whereas ongoing maintenance therapy, biochemical monitoring, and selected survivorship visits can often be organized through hybrid routes [5,6,11]. HCT and CAR T-cell therapy remain among the least virtualizable components of hemato-oncology, as their safety depends on donor and product logistics, protected inpatient capacity, transfusion support, infection screening, and specialized multidisciplinary coordination [28-30].

Transplantation and cellular therapy introduced another layer of logistical challenges, because donor availability, product collection, cryopreservation, protected inpatient and critical-care capacity, and infection status had to be secured before treatment could proceed [28,30,31]. EBMT and other transplant-focused recommendations supported screening of recipients and donors, reduction of avoidable hospital exposure, consideration of product cryopreservation in selected contexts, and individualized postponement when the oncologic risk of delay was acceptable [30,31]. At the activity level, the pandemic effect on transplantation was measurable: the EBMT activity survey by Passweg et al. reported 45,364 HCT procedures in 41,016 patients across 690 centers in 2020, compared with 48,512 HCT procedures in 43,581 patients in 2019, marking the first recorded decrease in EBMT transplant activity after decades of growth [28,32].

**Figure 2:**
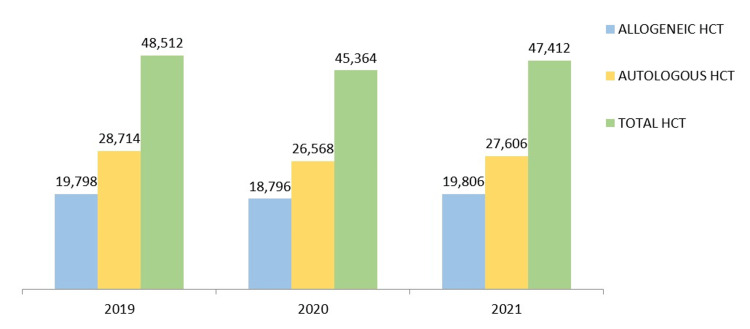
European HCT activity before and after the first pandemic shock. The grouped bars show allogeneic, autologous, and total HCT activity in 2019, 2020, and 2021, based on EBMT activity survey data reported by Passweg et al. [28,29,32]. The figure was created by the authors for this review using published EBMT data and Microsoft Excel 2016 for Windows (Microsoft Corp., Redmond, WA). EBMT, European Society for Blood and Marrow Transplantation; HCT, hematopoietic cell transplantation

The subsequent EBMT activity report showed partial recovery in 2021, with 47,412 HCT procedures reported in 43,109 patients across 694 EBMT-reporting centers, supporting a phased interpretation in which 2020 was dominated by access protection and deferral logic, whereas later pandemic phases increasingly reflected recovery, prioritization, and adaptation rather than simple suspension [28,29]. Because HCT depends on donor logistics, protected inpatient capacity, specialized staff, infection-control assurance, transfusion support, and multidisciplinary coordination, high-complexity hemato-oncology care was affected differently from outpatient follow-up and remote review. Changes in transplant activity therefore provide a useful indicator of the initial vulnerability and subsequent recovery of high-complexity hemato-oncology infrastructure, particularly in European and EBMT-associated settings [28,29,32]. Figure 2 summarizes the initial decline in EBMT-reported activity and the subsequent partial recovery phase.

Patient-flow redesign, day-hospital circuits, and inpatient access

The redesign of patient flow was one of the most visible organizational changes, because before the pandemic patients frequently entered hemato-oncology spaces through crowded outpatient clinics, day-hospital waiting areas, EDs, or inpatient admission routes that were not primarily constructed around respiratory infection separation [5,7]. By normalizing pre-visit triage and exposure-reduction measures, the pandemic changed the function of the outpatient clinic, transforming it from the default site for most clinical contact into one possible endpoint of a prior triage process. Hospital attendance became increasingly dependent on whether the encounter required physical examination, treatment administration, transfusion, urgent review, imaging coordination, procedures, or complex decision-making [5,7,8].

Day-hospital units also became more structured, as appointment spacing, pre-treatment laboratory review, isolation of patients with respiratory symptoms, fewer accompanying persons, and shorter waiting or chair times were introduced initially to reduce viral exposure [5,7]. These measures should be interpreted as operational adaptations rather than as uniformly validated quality-improvement interventions, but they remain relevant because many sequential day-hospital processes, including laboratory validation, drug preparation, infusion, transfusion compatibility testing, and discharge instructions, can be coordinated earlier to reduce avoidable waiting and exposure [5,7]. In the Kazakhstan dual-center study, Yerdenova et al. evaluated hospitalization rates, referral sources, outcomes, bed-days, residence, and diagnostic groups, and concluded that access to hemato-oncology services had been negatively affected during the pandemic, with altered referral dynamics and higher mortality signals [18]. Although these data are context-dependent and cannot be generalized mechanically to all European or North American centers, they make visible a clinically plausible pattern: when ordinary outpatient and referral pathways fail, specialist hemato-oncology services may receive patients later, through more urgent or indirect routes, with less opportunity for planned diagnostic sequencing [18].

Inpatient care required a different balance, because hemato-oncology cannot avoid admission when patients require induction chemotherapy, management of severe cytopenias, febrile neutropenia, tumor lysis syndrome, bleeding, urgent transfusion support, HCT, CAR T-cell therapy, or treatment of complex infections [5,15,27,30,31]. Flow reorganization should therefore be interpreted as a selective quality-improvement opportunity, not as a justification for restricting necessary access. Infection-control circuits, appointment discipline, separation of patients with respiratory symptoms, and pre-visit triage should remain, whereas excessive barriers to access, delayed diagnostic workup, and administrative gatekeeping that prevents timely review of symptomatic patients should not.

Telemedicine and hybrid care

Telemedicine became one of the most durable pandemic adaptations, but its value in hemato-oncology depends on patient selection rather than on remote access alone [11,16,33]. Broader oncology telemedicine literature documented large-scale tertiary-center feasibility, while also emphasizing that virtual care should not replace physical examination, procedures, urgent evaluation, or acute toxicity assessment when these are clinically required [34,35]. Berlin et al. reported 22,085 virtual-care visits across a tertiary cancer center during the early pandemic, with virtual care representing 68.4% of daily visits after implementation compared with 0.8% before launch, supporting the feasibility of rapid deployment at scale in a high-volume oncology setting [34]. In hematologic malignancy care specifically, Binder et al. described a rapid transformation at the Sidney Kimmel Cancer Center, where telehealth increased from approximately 2% to 50% of visits within weeks, illustrating how quickly virtual infrastructure could be deployed when ordinary clinic flow became unsafe or inefficient [8]. The strongest lesson from this literature is that virtual care cannot replace hemato-oncology, but it can prevent unnecessary hospital attendance when the clinical question is suitable, particularly for stable CML on tyrosine kinase inhibitor therapy, CLL under observation, indolent lymphoma follow-up, post-treatment survivorship, review of stable laboratory results, predictable toxicity checks after outpatient regimens, prescription renewal, or counseling about vaccination or infection symptoms when a clear safety net is available [8,11,16].

In contrast, new diagnosis, suspected progression, B symptoms, new lymphadenopathy requiring examination, rapidly worsening cytopenias, febrile neutropenia, transfusion need, severe mucositis, bleeding, dyspnea, neurologic symptoms, marrow evaluation, or treatment initiation should remain in-person or at least rapidly convertible to in-person care [5,6,15]. In the Barcelona real-life hematology study, Condom et al. reported reductions in outpatient initial visits and chemotherapy sessions of 55% and 19%, respectively, while phone visits increased by 581% during the early pandemic period [36]. In allogeneic transplant follow-up from Northern Italy, Lupo-Stanghellini et al. reported that 29 of 50 patients with chronic graft-versus-host disease were managed by phone, and that 125 of 203 visits for non-respiratory problems were conducted remotely [37]. Nawas et al. further described 27 telehealth visits in 25 patients undergoing HCT evaluation, supporting feasibility in selected transplant-related encounters [38]. Taken together, these findings support a selective model in which remote review is used for suitable follow-up or information-based encounters, while treatment continuity remains protected. This distinction is clinically important because pre-pandemic evidence from Borel et al., involving 380 patients with DLBCL receiving R-CHOP, linked treatment adherence to survival, reinforcing that efforts to reduce exposure should not compromise the delivery of effective therapy [39].

The Princess Margaret Cancer Centre experience provides a hematology-specific quantification of this shift. Suleman et al. reviewed 9,295 patients with HMs from October 2019 to March 2021 and found that 12.1% had at least one virtual visit, with virtual care absent before March 2020, rising to 36% from March to August 2020, and persisting at 30% from September 2020 to March 2021 [33]. Virtual visits were concentrated particularly in lymphoma and myeloma clinics, which accounted for 36% and 46% of virtual visits, respectively, and patients who had virtual visits were more likely to request virtual follow-up than those seen in person, supporting the acceptability of remote care in selected hematology encounters [33]. These findings are consistent with the broader virtual-care literature in HMs, which suggests that remote review can be useful when the encounter is based mainly on laboratory interpretation, symptom review, counseling, or follow-up planning rather than examination, procedures, or treatment administration [11,33]. In this sense, hybrid care may partially change the geography of hemato-oncology by allowing selected patients to combine local laboratory testing with remote specialist interpretation, provided that symptom instability, examination needs, and treatment changes are explicitly screened [11,33,40].

Equity remains an important challenge of hybrid care. Older patients, patients with poor internet access, limited digital literacy, language barriers, reduced capacity to report symptoms remotely, or a need for physical examination may be disadvantaged if remote care becomes a default route rather than an option selected according to clinical suitability and patient capacity [11,16,41]. The durable standard should therefore be hybrid care by indication, not remote care by convenience [11,33]. Neparidze et al. provide a hematology-specific equity signal: in a United States cohort of 18,924 active patients with HMs and 884,504 analyzed visits, including 2,394 Black patients and 16,530 White patients, telemedicine uptake was significantly higher among White patients than among Black patients across all diseases and treatment categories [41]. Virtual care may reduce travel and exposure for some patients, but unless access, technology, language, and socioeconomic barriers are actively monitored and addressed, it may also preserve or amplify pre-existing inequalities [41].

International comparisons place the hemato-oncology experience within a broader health-system transformation rather than an isolated specialty-specific response [42,43]. In a 20-country cross-sectional study of 1,370 primary care physicians, Kerr et al. reported that telephone consultation availability increased from 73.1% before the pandemic to 90.3% during the pandemic, video consultation availability increased from 12.8% to 52.3%, and chat consultation availability increased from 33.7% to 42.3% [42]. These data are not hemato-oncology-specific, but they provide useful context for understanding why remote triage and hybrid follow-up became plausible components of post-pandemic hematology pathways. Wosik et al. similarly described telehealth transformation as a broader reconfiguration of care delivery during COVID-19, involving workflow, technology, staffing, reimbursement, and patient-access changes rather than only a change in consultation format [43].

Supportive care, transfusion, vaccination, and antiviral routes

Supportive care was both disrupted and restructured during the pandemic. Shah et al., in a global systematic review and meta-analysis of 245 studies from 46 countries, included supportive and palliative care among the cancer-service domains affected by pandemic-related disruption, alongside screening, diagnosis, diagnostic procedures, and treatment [2]. This disruption is particularly concerning for hemato-oncology because supportive care is not ancillary: transfusions, antimicrobial prophylaxis, growth factor support, central venous access management, immunoglobulin replacement in selected patients, and rapid treatment of febrile neutropenia are often prerequisites for delivering disease-directed therapy safely [5,6,15]. By accelerating pre-treatment planning, the pandemic made CBC review before arrival, scheduled transfusion slots, telephone toxicity assessment, earlier outpatient antimicrobial decisions, and clear instructions for fever or respiratory symptoms part of the patient-flow architecture [5,7]. In selected settings, granulocyte-colony stimulating factor (G-CSF) use was considered more actively to reduce neutropenia duration and emergency presentations, although this remained disease- and regimen-dependent rather than universal [5,15].

Although vaccination changed risk management, it did not eliminate vulnerability. In the CAPTURE prospective cohort, Fendler et al. evaluated 585 patients with cancer after two vaccine doses and reported lower seroconversion among patients with HMs than among patients with solid tumors, 59% versus 85%, together with more frequent undetectable neutralizing antibody titers and lower median neutralizing titers against wild-type SARS-CoV-2 and variants of concern [44]. This finding is consistent with systematic review and meta-analysis data showing reduced serologic response in patients with hematologic cancers, particularly B-cell malignancies such as CLL and lymphoma, and in patients exposed to B-cell-depleting therapy [13,45,46]. Sakuraba et al. estimated a serologic response after two vaccine doses of 63.7% in hematologic cancers compared with 94.9% in solid tumors, while Vijenthira et al. showed markedly impaired vaccine responsiveness during active anti-CD20 therapy [45,46].

As pandemic care matured, antiviral pathways became increasingly important. ECIL-10 post-pandemic recommendations support early antiviral treatment with nirmatrelvir/ritonavir or remdesivir in high-risk symptomatic patients, while also highlighting drug-drug interactions, timing of therapy, and individualized decisions regarding delay of intensive chemotherapy, conditioning, or T-cell-directed therapy during active infection [24].

The broader supportive-care message is that hemato-oncology services should not separate anticancer treatment from infection logistics. A patient scheduled for chemotherapy, immunotherapy, transplant conditioning, or cellular therapy should be supported by a route that integrates disease status, SARS-CoV-2 and seasonal respiratory virus risk, vaccination history, antiviral eligibility, transfusion needs, and local access to urgent evaluation, without allowing infection management to cause avoidable delay in clinically necessary cancer treatment. The infectious-disease component of this model is supported by post-pandemic COVID-19 guidance, EBMT recommendations for HCT and CAR T-cell therapy, and evidence on prolonged viral replication in patients with HMs [24,25,30].

What changed, what was abandoned, and what should persist

Some measures should persist, some should be abandoned, and some should remain only in modified form. Broad cancellation of services, generalized delay of diagnostic procedures, and administrative obstacles to urgent specialist review belong in the abandoned category, because cancer-service disruption and treatment delay were associated with measurable oncologic risk [2,19]. Excessive restrictions and disrupted access were also visible at the health-system level and in hemato-oncology-specific service data, including the marked reduction in United States screening volumes reported by DeGroff et al. and the altered access routes described by Yerdenova et al. in hemato-oncology services in Kazakhstan [17,18]. By contrast, structured triage, safeguarded diagnostic capacity, appointment discipline, separate respiratory circuits, and coordinated day-hospital scheduling represent defensible post-pandemic organizational safeguards [5,7]. Selective virtual review of stable patients and local laboratory monitoring are supported by hematology-specific virtual-care literature, while rapid antiviral access is supported by post-pandemic ECIL-10 guidance [8,11,16,24,33].

To avoid overextending evidence from general oncology to hemato-oncology, the synthesis distinguishes between direct HM- or HCT-specific evidence and broader oncology or health-system evidence used only for contextual interpretation. To translate these general principles into service-level examples, Table 1 summarizes selected country-level signals relevant to hemato-oncology care routes, distinguishing disruption signals, pandemic adaptations, and likely post-pandemic organizational legacies.

**Table 1:** Selected country-level service delivery signals relevant to hemato-oncology care pathways. This table includes only service-delivery signals directly documented in the cited sources. The final column represents the authors’ conservative narrative interpretation. General oncology and cancer-screening data are used only as contextual evidence and are explicitly distinguished from HM-specific or HCT-specific evidence. Source mapping by country: United Kingdom [9,10,31]; United States [8,17,38,41]; Italy [7,37]; Spain [22,25,36]; Canada [33,34]; Kazakhstan [18]. HCT, hematopoietic cell transplantation; HMs, hematologic malignancies; HSCT, hematopoietic stem cell transplantation; SARS-CoV-2, severe acute respiratory syndrome coronavirus 2

Country	Main disruption signal	Pandemic adaptation	Likely post-pandemic legacy
United Kingdom	-Cancer-delay modeling estimated excess mortality or life-years lost related to pandemic-associated diagnostic and urgent-referral delays.	-Prioritization of time-sensitive cancer pathways and HSCT activity under pandemic constraints. -Rapid guideline-based service organization for HSCT. -Rapid guidance was developed for HSCT services during COVID-19.	-Protected diagnostic/referral capacity should be preserved for time-sensitive cancer presentations. -HSCT programs require pre-defined prioritization and continuity plans during viral respiratory surges.
United States	-Early cancer-screening disruption was documented, including major reductions in breast and cervical cancer screening. -Rapid telehealth expansion was described for patients with HMs. -Racial differences in telemedicine uptake were reported among patients with HMs.	-Rapid telehealth and digital-care deployment for selected hematology/oncology encounters. -Use of telehealth for selected HCT-related evaluations. -Recognition that virtual-care implementation requires equitable monitoring. -Telehealth evaluation was reported in selected HCT candidates.	-Selected telehealth can remain useful for suitable follow-up encounters. -In-person care should be preserved for physical examination, procedures, treatment delivery, and urgent assessment. -Telemedicine programs should monitor access barriers and inequities.
Italy	-A tertiary hematology center in Lombardy described continuity measures during severe early pandemic pressure.	-Protected hematology pathways and continuity measures for vulnerable patients. -Selective remote follow-up for defined transplant-related encounters. -Remote follow-up was described in selected allogeneic transplant recipients, including patients with chronic graft-versus-host disease.	-Protected circuits for fragile hematology patients should remain available during respiratory-infection surges. -Remote follow-up may be retained for selected stable transplant-related encounters.
Spain	-Real-world hematology activity in Barcelona showed reduced outpatient initial visits and chemotherapy sessions, with a marked increase in phone consults. -Prolonged SARS-CoV-2 replication was documented in a subset of hospitalized patients with HMs.	-Expanded phone-based follow-up in hematology services. -Infection-aware management of vulnerable hematologic patients. -Attention to prolonged viral replication in immunocompromised patients.	-Hybrid follow-up may be appropriate for selected hematology encounters. -Persistent infectious-risk stratification remains necessary in vulnerable HMs. -Pathways should account for possible prolonged viral replication.
Canada	-Large-scale virtual-care implementation was reported in a tertiary cancer center. -Virtual-care use was reported in 9,295 patients with HMs at Princess Margaret Cancer Centre. -In the HM cohort, virtual care was absent before March 2020, increased rapidly during the early pandemic, and persisted in later months.	-Rapid virtual-care deployment in tertiary oncology. -Virtual visits for selected HM follow-up encounters. -Remote review of suitable information-based encounters.	-Selected virtual follow-up is feasible and acceptable in oncology and HMs. -The evidence does not justify universal virtualization of hemato-oncology care. -Physical assessment, treatment delivery, procedures, and urgent clinical change still require in-person capacity.
Kazakhstan	-In 2020-2021, fewer discharges, a higher death proportion, more frequent emergency/ambulance admissions, and increased transfers from other hospitals were reported.	-Specialist hemato-oncology centers received more patients through urgent or indirect access routes during disrupted ordinary pathways.	-Hemato-oncology services require protected referral channels and resilient access routes during system stress. -The findings are context-specific and should not be generalized mechanically to other health systems.

Beyond the country-level signals summarized in Table 1, the strongest retained adaptation is risk-stratified care rather than telemedicine alone. Before the pandemic, many pathways were organized around periodic hospital attendance. During the pandemic, they were reorganized around infection exposure and resource scarcity. After the pandemic, routing should be organized around clinical necessity, with in-person care preserved for situations in which examination, procedures, treatment administration, severe toxicity, diagnostic uncertainty, or urgent decision-making make physical attendance valuable [5,8,11,16].

A second retained adaptation is the separation of flows, because immunocompromised patients should not share uncontrolled clinical space with patients with acute respiratory symptoms when avoidable, and hematology units should maintain seasonal respiratory triage, rapid testing capacity, and internal rules for febrile or symptomatic patients even outside declared pandemics [5,7,24].

A third retained adaptation is the more deliberate use of day-hospital time. Patients with HMs often move through sequential processes, including laboratory sampling, result validation, drug preparation, physician approval, infusion, transfusion compatibility testing, prescription renewal, and discharge instructions. Pandemic-era scheduling made these steps more visible as targets for coordination, even though the effect on waiting time and unit capacity was not uniformly measured across studies [5,7,18].

A fourth retained adaptation is the recognition that patients differ not only by diagnosis, but also by current care phase. A newly diagnosed patient with diffuse large B-cell lymphoma (DLBCL), a patient with CLL under observation, and a patient with acute leukemia do not have the same access thresholds, tolerance for delay, or need for in-person review [5,15,27]. Similarly, a post-transplant patient with chronic graft-versus-host disease may be suitable for selected remote follow-up under defined conditions, whereas a CAR T-cell therapy recipient with fever requires rapid infectious and toxicity-oriented assessment rather than routine virtual review [24,30,37].

Proposed post-pandemic hemato-oncology model

A practical post-pandemic model can be built around four decision gates. These gates should be interpreted as overlapping decision domains rather than as rigid sequential steps. Treatment-prioritization evidence supports the first two gates, diagnostic urgency and treatment intent [5,6,15,27]. Virtual-care literature supports the encounter-value gate [8,11,16,33]. ECIL-10 and EBMT guidance support the immune and infectious-risk gate, particularly for patients with HMs, HCT recipients, and patients undergoing CAR T-cell therapy [24,30].

The first gate is diagnostic urgency: suspected acute leukemia, aggressive lymphoma, marrow failure, severe cytopenia, rapidly progressive lymphadenopathy, B symptoms, tumor lysis risk, or neurologic compromise should bypass routine scheduling and enter a protected urgent diagnostic pathway [5,6,15,27]. The second gate is treatment intent: curative-intent therapy and high-benefit disease control should not be delayed for administrative convenience, whereas low-benefit or elective interventions can be scheduled with more flexibility when infection risk, patient frailty, or system pressure is high [5,6]. The third gate is immune and infectious risk: recent anti-CD20 therapy, hypogammaglobulinemia, profound lymphopenia, weak vaccine response, active infection, transplant history, CAR T-cell therapy, profound neutropenia, active steroid exposure, or uncontrolled disease should lower the threshold for testing, antiviral treatment, protected in-person review, or individualized treatment timing [13,24,25,30,44,46]. The fourth gate is encounter value, meaning that remote review may be preferred when the patient is clinically stable and an in-person visit is unlikely to change management, whereas in-person review remains necessary when examination, procedure, infusion, transfusion, imaging coordination, or urgent clinical judgment is required [5,8,11,15,16,33].

**Figure 3:**
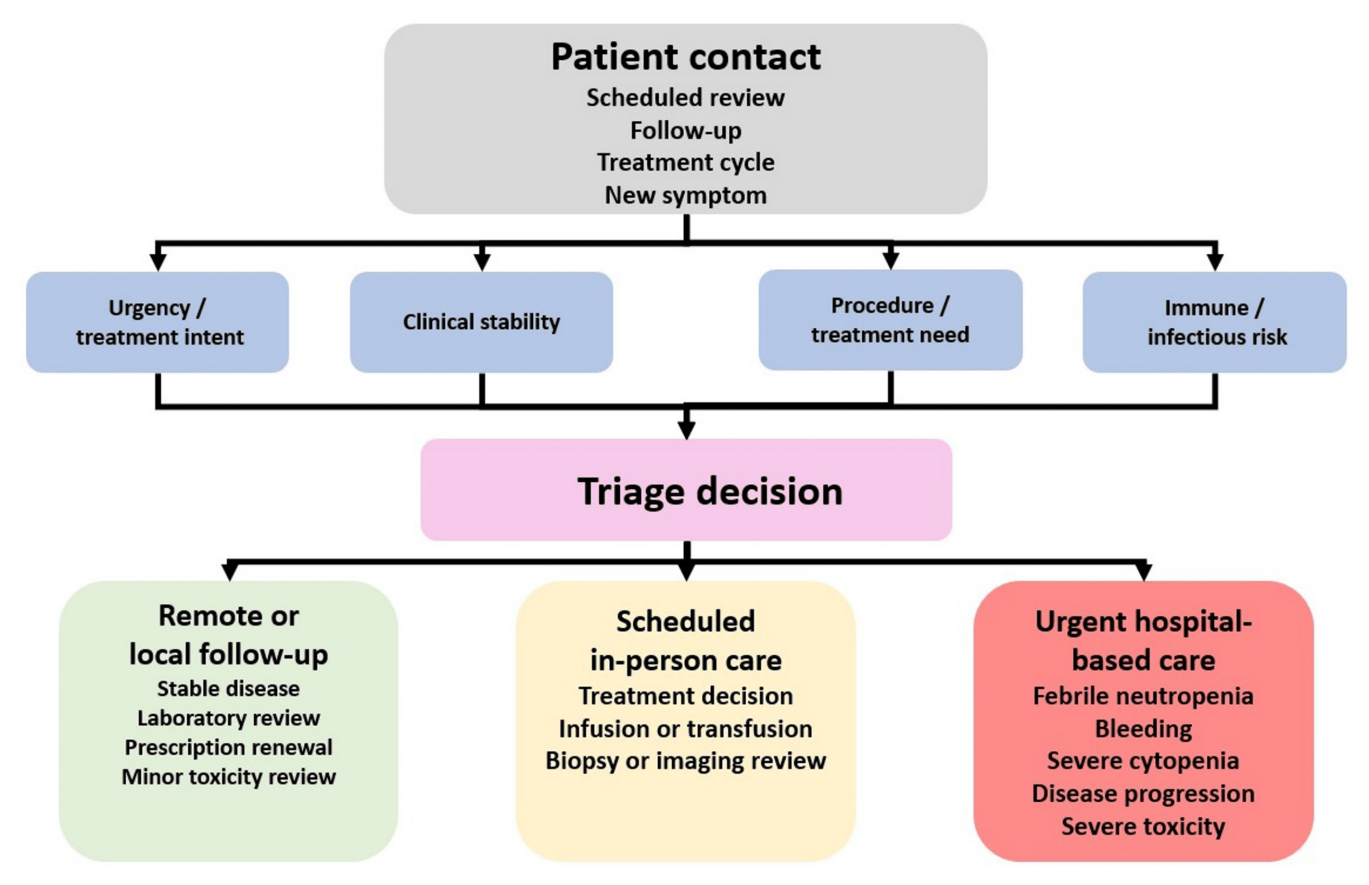
Proposed selective hybrid-care model for post-pandemic hemato-oncology. The figure represents an author-generated conceptual framework based on narrative synthesis and has not been independently validated. It is intended as an illustrative model and should be adapted to local resources and patient-level factors. Created using Microsoft PowerPoint 2016 for Windows (Microsoft Corp., Redmond, WA)

Figure 3 translates these four decision gates into a proposed selective hybrid-care model, in which the route of care is determined by diagnostic urgency, treatment intent, immune and infectious risk, and encounter value, rather than by a default preference for either in-person or remote care.

Limitations

The limitations of this review reflect its narrative design, because the pandemic literature combines registry data, modeling studies, institutional experience, expert consensus, health-system analyses, and disease-specific recommendations rather than a uniform body of comparable trials. The international indicators used here also have different denominators and scopes: the cross-country virtual consultation data describe primary care availability rather than hemato-oncology-specific utilization [42], EBMT activity reports primarily capture European and EBMT-associated transplantation and cellular therapy activity [28,29], and global cancer disruption estimates include many solid-tumor services that only partially overlap with care for patients with HMs [2]. Consequently, the conclusions presented here should be read as a clinically oriented synthesis of pathway changes, not as a quantitative estimate of the global magnitude of disruption, recovery, or benefit.

Several limitations arise from the narrative design of this review. Although the search strategy, thematic axes, source-retention principles, and evidentiary-weighting approach were described to improve transparency, this review was not conducted as a PRISMA (Preferred Reporting Items for Systematic Reviews and Meta-Analyses)-based systematic review and did not include a formal risk-of-bias assessment or quality appraisal of individual studies. Consequently, the synthesis may be affected by selection bias, publication bias, uneven availability of hemato-oncology-specific evidence across care domains, and author-level judgment in deciding which sources were most relevant to care delivery, patient flow, and post-pandemic organizational adaptation. In addition, broader oncology and health-system evidence was used in selected areas where direct hemato-oncology data were limited, which may reduce disease-specific precision. The conclusions should therefore be interpreted as a clinically oriented narrative integration of the available evidence and service-delivery experience rather than as a reproducible systematic evidence appraisal or a validated care framework.

Post-pandemic practice also continues to evolve, as circulating variants, vaccination coverage, antiviral availability, testing capacity, electronic health infrastructure, day-hospital capacity, rural referral patterns, and local workforce constraints differ across countries and institutions. The practical value of this review therefore lies in identifying transferable principles, including protected diagnostic access, risk-stratified attendance, hybrid follow-up for selected patients, rapid infectious-risk pathways, and preserved high-complexity in-person capacity, while acknowledging that the specific configuration of these principles must remain local.

## Conclusions

The COVID-19 pandemic should be interpreted in hemato-oncology not only as an infectious threat, but also as a stress test of a highly hospital-dependent care model. It exposed vulnerabilities in diagnostic access, day-hospital organization, inpatient capacity, transplantation and cellular therapy logistics, supportive-care delivery, and follow-up pathways built around repeated in-person contact. The durable lesson is not to preserve crisis restrictions or to expand virtual care indiscriminately, but to retain a disciplined routing model in which disease tempo, treatment intent, immune status, infection risk, procedure dependence, and clinical stability determine the route of care. Post-pandemic hemato-oncology should preserve protected diagnostic and treatment access, structured triage, separated respiratory pathways, coordinated day-hospital scheduling, rapid infectious-risk assessment, early antiviral access for high-risk patients, and selective hybrid follow-up for stable encounters. At the same time, diagnostic delay, administrative gatekeeping, reduced supportive-care access, and undertreatment of aggressive or potentially curable disease should be rejected as unacceptable crisis residues. The central objective is not less hemato-oncology care, but better-routed care.
